# Nutritional status of children under-five in rural Mozambique: the role of phytate intake and mineral bioavailability

**DOI:** 10.1186/s41043-026-01247-4

**Published:** 2026-02-14

**Authors:** Ieva Sateikaite, Yasaman Samaei, Maida Khan, Irene Carvalho, Claudia E. Lazarte

**Affiliations:** 1 Division of Food and Pharma, Department of Process and Life Science Engineering, Faculty of Engineering, Lund University, Lund, Sweden; 2 Departamento de Engenharia Química, Faculdade de Engenharia, Universidade Eduardo Mondlane, Maputo, Mozambique; 3 National Food Institute, Technical University of Denmark (DTU), Kongens Lyngby, Copenhaguen, Denmark

**Keywords:** Nutritional status, 24-h food photography recall, Mozambique, Phytate, Mineral bioavailability

## Abstract

**Background:**

Globally, undernutrition is responsible for over 3.5 million deaths among children under the age of 5. In Mozambique, it remains a major contributing factor to the high rates of child mortality and illness. Essential micronutrients like zinc, iron, and calcium play a vital role in healthy growth and development. However, their absorption is hindered by anti-nutrients such as phytates, which chelate these minerals and reduce their bioavailability. The extent of this inhibitory effect largely depends on the molar ratios of phytate to the respective minerals.

**Objective:**

To assess the dietary patterns and quality of diets consumed by young children; with a primary focus on micronutrients adequacy, phytate intake and the implications on mineral bioavailability.

**Methods:**

Anthropometric measurements and dietary assessment of 47 children, aged 2 to 5 years, accompanied by their mothers. The method Food Photography 24-h Recall (FP24hR) was used for three consecutive days to evaluate their nutrient intake, which included macronutrients, 15 micronutrients and phytate intake. The estimated mineral bioavailability of the diets was determined based on the phytate-to-mineral molar ratios.

**Results:**

Stunting was found in 32% of children and waste in 6% of them, 2% of children were underweight, and 13% were overweight. Children were found to be at risk of calcium, zinc, vitamins A, E, B12 and folate deficiencies. The molar ratio phytate-to-Zinc (phy:Zn) was between 22.3 and 31.2; phytate-to-Iron (phy:Fe) was between 7.2 and 16.3 and phytate-to-Calcium (phy:Ca) was between 0.34 and 0.53. All the ratios exceeded the recommended values phy:Zn < 15, phy:Fe < 1, and phy:Ca < 0.24, which indicates that phytate in the children’s diet can negatively affect the bioavailability of zinc, iron and calcium.

**Conclusion:**

The high prevalence of stunting among children in this study highlights a persistent issue of chronic undernutrition in the region. The elevated phytate-to-mineral molar ratios suggest a reduced mineral absorption, which is a contributing factor to growth failure in children. These findings underscore the urgent need for targeted nutrition policies in Mozambique to address both dietary quality and phytate content.

## Background

Child malnutrition is a global public health problem with serious implications for child survival. It hinders childrens cognitive and physical development while also reducing the economic productivity of both individuals and communities [1]. Adequate nutrition is especially crucial during the first 1,000 days of life, from conception through pregnancy and the first two years after birth, represent a uniquely critical window for a child’s growth, brain development, and long-term health. During this period, tissues and organs form at a rapid pace, making adequate nutrition essential. While macronutrients provide the energy needed for growth, micronutrients play equally vital roles in cognitive development, immunity and healthy growth. [2]. A systematic review examining iron and zinc intake during the first 1,000 days of life found that interventions providing these micronutrients during this critical window effectively reduced the risk of deficiencies [3].

Children’s nutritional status can be assessed by anthropometric measurement (z-scores for wasting, stunting and underweight) as they are key indicators of malnutrition [4]. Undernutrition is linked to nearly half of all deaths among children under 5, particularly in low- and middle-income countries (LMICs). In 2022 the WHO, estimated that 149 million (22.3%) children under the age of 5 were stunted, 45 million (6.8%) suffered from wasting, and 37 million were overweight or obese [5],[6].

A healthy diet for children is a diet that provides adequate quantity and quality of nutrients that can cover the average nutrient requirements for their specific age group. Protein, fat, and carbohydrates are essential in the diet of a growing child, each playing critical roles in supporting healthy growth and development [7]. Nutrient inadequacy may result in increased risk of chronic disease as well as impaired growth [8]A lack of a balanced diet can result in micronutrients deficiencies, even within an omnivorous diet, particularly in the context of maternal and child undernutrition and food insecurity in LMICs [9]. Deficiencies in iron, zinc, vitamin A, vitamin D, and calcium are prevalent in many countries. Iron-deficiency anemia is the most common nutritional deficiency in the world affecting approximately 40% of children under five [10]. According to UNICEF, in 2013, vitamin A deficiency affected one third of children under 5, with the highest rate in sub-Saharan Africa (48%) [11]. Zinc is essential for children’s growth and development, supporting immune function, wound healing, and sensory processes such as taste and smell [12]. Children in South Asia, sub-Saharan Africa and Central America are at the risk of inadequate zinc intake [13]. Additionally, Calcium and vitamin D are crucial for bone structure, strength, and development, they play pivotal role in preventing rickets [14].

The diet traditionally consumed in rural Mozambique as previously described is predominantly plant-based [15], and mainly composed of cereals and starchy staples. This type of diet is poorly diversified with almost 80% of dietary energy derived from cereals and starchy roots. Consequently, it is extremely low in micronutrients and protein, and fails to provide sufficient energy to meet the recommended dietary requirements [15]. Additionally, plant-based foods are rich in phytate, this compound exhibits a strong capability to chelate divalent metal ions such as iron, zinc, calcium, and manganese, forming insoluble salts. Thus, the presence of phytate in the diet can inhibit the absorption and bioavailability of these nutrients [16]. The inhibitory effect of phytate on minerals is largely dependent upon phytate-to-mineral molar ratios. The recommended phytate-to-zinc (phy:Zn value is below 15; phytate-to-calcium (phy:Ca) is below 0.24, and phytate-to-iron (phy:Fe) is below 1. Ratios above these limits indicate that the bioavailability of minerals is significantly affected by phytate content in the diet [, 17, 18, 19].

To date, there has been a lack of reliable data on dietary patterns and adequacy, as well as on mineral bioavailability of diets in most African countries, and how these factors may be contributing or linked to the food insecurity in the region. The aim of this study is to provide reliable data on anthropometric indicators, dietary patterns, and micronutrient adequacy in children under five in the rural area of Mozambique, Bobole in the district Marracuene, Maputo. Additionally, the study examined the phytate content and the estimated bioavailability of key minerals, including iron, zinc and calcium, in the children’s diets. This study also explores how these factors are related to food environments and food insecurity, which could serve as a starting point to inform policy makers about the nutritional situation of children in a rural area in Mozambique. The methods used in this study can be replicated in other areas to gain a broader understanding of the nutrition situation in Mozambique.

## Methods

### Study design and food photography 24-h recall (FP24hR)

**Fig. 1:**
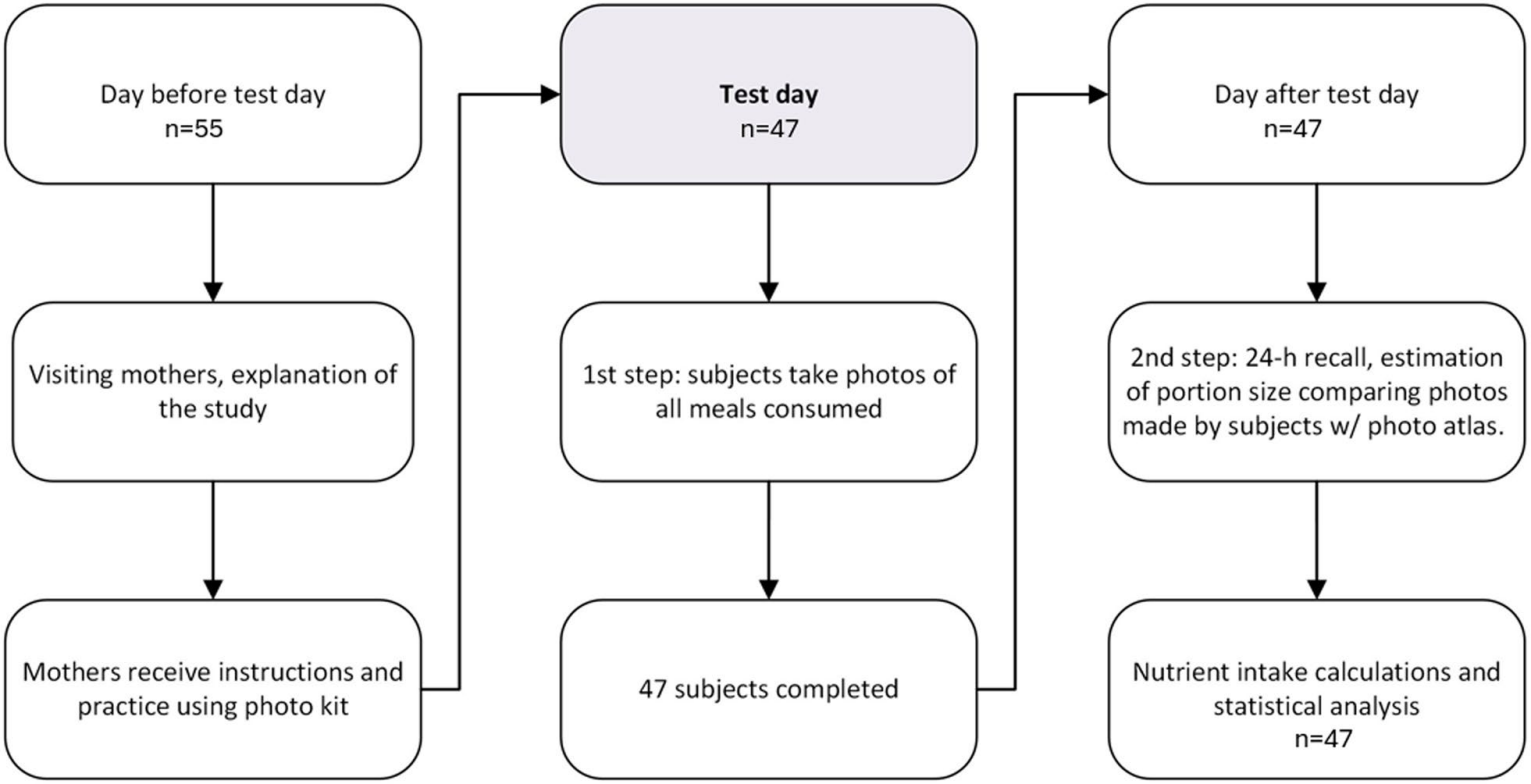
Design of the study, where test day is one day of data collection

The design of the study and the FP24hR method were adapted from a previously validated study conducted in a rural population of Bolivia [20]. The study design had two steps; first mothers took digital photographs of all foods consumed by their children over a period of 24 h. In the second step, the following day, an interview was carried out using a 24-h recall questionnaire, in which mothers were asked to estimate and report on the quantities of food and drinks consumed by their children the day before. They were also asked to provide detailed information on ingredients quantities and methods of food preparation. The digital photographs were used as a memory aid for better recalling all consumed foods and ingredients. Portion sizes were estimated by comparing food in the digital photographs with standard food photographs in the photo atlas. Figure 1 depicts the study design.

### Subjects

This study was performed in a rural area called Bobole, a region located about 60 km north of Maputo, the capital of Mozambique. Bobole belongs to the Marracuene district and has a population of about 6000 inhabitants (1400 families). Bobole is the biggest area within the district and is populated by 994 families. The area was intentionally selected as a convenience sample for this study due to its commonly spoken languages (Portuguese and Shangaan) and its reasonable travel distance from Maputo, making it practical and feasible for the data collectors to ensure proper data collection.

The study was performed with children under 5 years of age; thus, their mothers were also included as active participants assisting in providing information on children’s diet. The participating families were kindly invited by the local chief of the community (about 90 families received information on the project), the chief and families were briefed on all the steps of data collection and approved the study protocol. We visited 55 families and showed in detail how the method should be conducted, from which 47 families agreed to be part of the study. The chief was present every day throughout the entire study period to help participants feel more comfortable and build trust. The mothers’ participation depended on their willingness to take pictures of all foods given to their children for three consecutive days, and to answer detailed questions on the preparation of these foods. We selected consecutive days in this dietary study to simplify scheduling and ensure complete and accurate data collection for this age group; however, this approach may over- or under-estimate habitual intakes. They all signed a letter of consent prior to the study period. The sample size of this study was calculated based on the results obtained in previous studies conducted by C.E. Lazarte et.al., 2012 [20] in Bolivia. Using z-scores with 95% confidence intervals to estimate iron, zinc and protein intake, the minimum required sample was determined to be 25, 27 and 41 children respectively. A total of 47 children and mothers completed the study. For a better interpretation of the results, the participants were divided into two sub-groups based on age 2–3 years (n = 19) and 4–5 years (n = 28). This division may have reduced the statistical power of the findings.

### Anthropometric measurements

The height and weight of each child was recorded using a meter stick and a digital electronic scale. The children were barefoot and wore light clothes during the time when measurements took place. Anthropometric indices were calculated using reference medians recommended by WHO and categorized by using standard deviation units: z-values. Calculations of the z-values -WHZ (weight-for-height), WAZ (weight-for-age), and HAZ (height-for-age) scores- were performed using the WHO Anthro (version v3.2.2.) program, developed especially for children ranging from 0 to 5 years of age [21, 22].

#### Questionnaire 24-h recall

The 24-h recall questionnaire was arranged following the format provided by Lazarte et al. [20]. It was translated into the official language, Portuguese, and validated with residents in the rural area to make sure that the questions were understandable and applicable. The questionnaire included questions addressing the names of foods eaten, names and amounts of ingredients, and ways in which food was prepared. It also included questions about whether any other snacks, drinks, vitamin/mineral supplements, or medicines were used in addition to what was seen in the photographs. The questionnaire was divided into the different times of the day that food consumption took place, such as breakfast, lunch, and dinner, as well as mid-morning and mid-afternoon snacks.

#### Photo kit

**Fig. 2:**
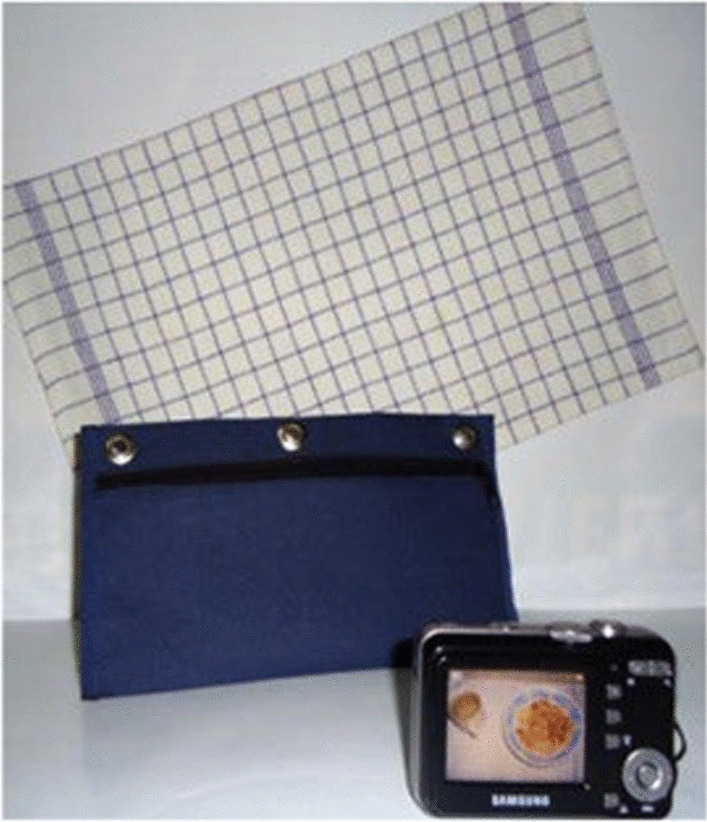
Photo kit: digital camera, camera case and marked table mat. (Photograph by Lazarte et.al. [20], reprinted with permission)

**Fig. 3:**
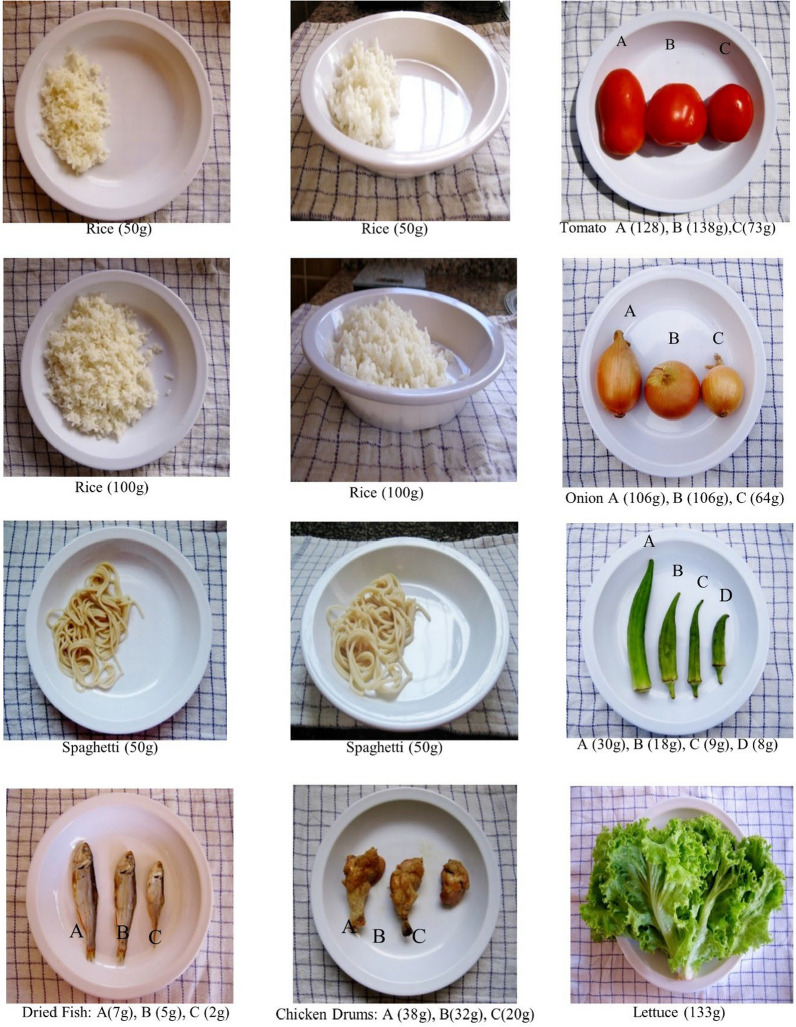
Example of food photographs in the photo atlas, showing portion sizes of cooked and raw food

A photo kit similar to the one used in our previous study, reference shown in Fig. 2, was provided, for the duration of the study, to the mothers in the study. It included a digital camera, a case, and a marked table mat with 1.5 cm grids to put the plate on top. The objective of the marked mat was to provide a standard background and a size reference. In this study, participants were also provided with standard plates, like those used to develop the food photo atlas (Fig. 3). At the time the photo kit was given to the mothers, interviewers also provided hands-on training on how to place the mat and plate, how to use the camera, and how to take photographs of the foods before and after consumption by the child during the test days.

#### Photo atlas

A photo atlas consisting of color photographs of common local foods was developed to assist interviewers and participants in estimation of portion sizes. Pictures of a total of 26 common foods in different portion sizes were taken to represent different quantities of foods. Overall, the atlas was composed of 95 food photos, divided into five groups: carbohydrates, fruits and vegetables, nuts and legumes, fish and poultry, and cooked leafy vegetables. Foods were grouped in this way due to the limited variety of food choices in the study area.

The food atlas was prepared following the example of a previously-used photo atlas [20]. The first photograph of each food for the photo atlas was taken at an angle of approximately 90º, and at a 50-cm distance above the plate. The second photograph was taken at an angle of approximately 45º, achieved by taking one step back from the plate. The second photo was necessary to display differences in portion sizes shown by the height of food on the plate. One-size plates were used for all the food in the photographs as the standard reference. The same plates were provided to the subjects and used as a standard reference for the study.

Figure 3 shows an example of photographs from the photo atlas. Foods illustrated in the photo atlas varied in portion size to most closely represent the portion sizes consumed by children. Portions were arranged in ascending order, with the smallest portion at the top and increasing in size toward the bottom.

The images were 78 × 72 mm in size, allowing six photos to be displayed on a single A4 sheet printed in color. The name and weight of the food were presented at the bottom of each photograph. Photos of raw ingredients such as fruit and vegetables, which vary in weight and size, were also included in the photo atlas. All raw ingredients for the photos were bought at the local food market in order to most closely depict foods actually consumed by the children. These photographs proved useful during the interview, when mothers described the ingredients in mixed dishes such as stews, relishes, sauces, and so on.

#### Food photographs as a memory aid the 24-h dietary recall

Children 2 to 5 years old were followed for a total of three consecutive days. The day before the actual test day, the interviewers, together with the local chief, visited the families in their homes to explain the procedure. Because the children were too young to answer the questions, the mothers of the children were asked to participate. If they agreed to participate, they were provided with the photo kit and verbal instructions, accompanied by demonstrations of how to use the camera to take suitable photographs of all meals given to the child. The mothers were encouraged to practice how to turn the camera on and off, and to take few photos, in order to make sure they understood the instructions.

**Fig. 4:**
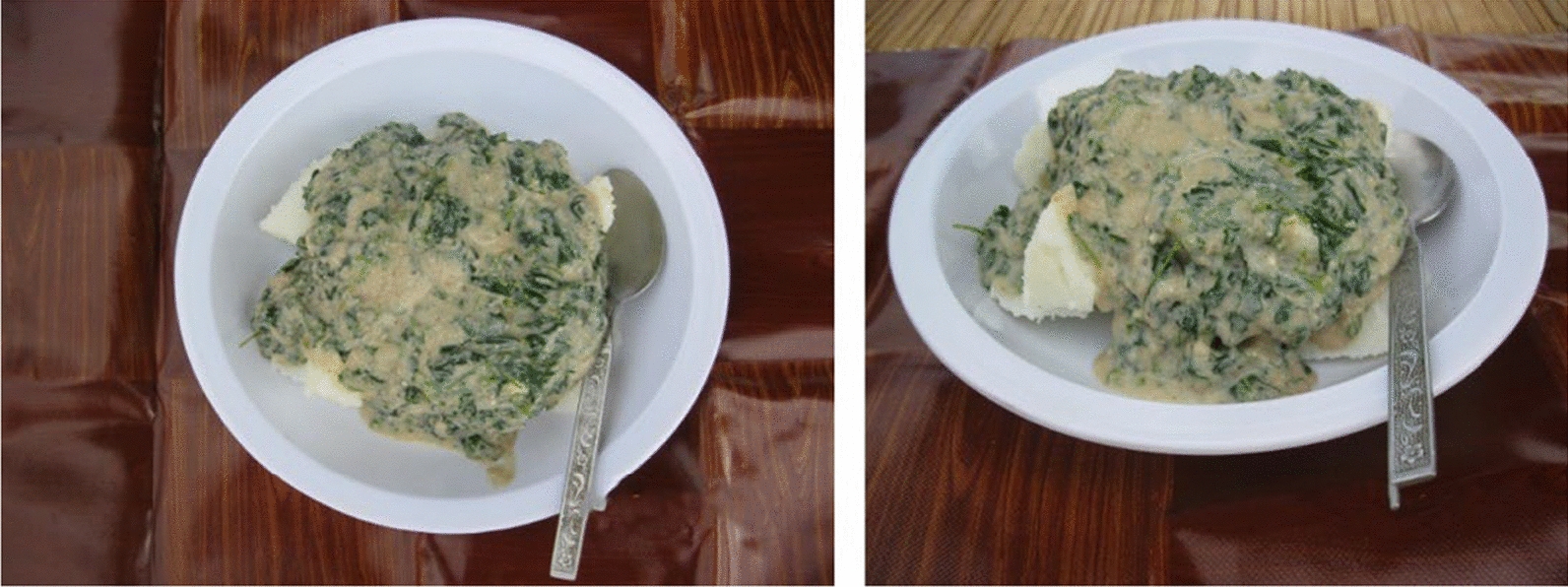
Example of food pictures taken by one of the participating mothers

In the first step of the study, the mothers took photographs of all the meals they gave to the children during the test day. Directions were given to place the plate containing the food on the table mat, and to take two photographs from the same 90º and 45º angles, in a similar way to the photos taken for the photo atlas. Mothers were asked to take another photograph of the plate if there were any leftovers. The reason for taking two photographs at different angles was to determine features of its appearance which might influence perception of quantity in the photographs. Features included the height and area of food pieces as well as the area and depth occupied by food on the plate. This contributed to a more accurate estimation of food portion sizes. Figure 4 shows an example of photographs taken by one of the participating mothers.

In the second step, on the following day, the interviewer asked the mothers to recall and estimate the precise foods and beverages their children had consumed during the previous day. Portion size estimation was done by comparing the digital photographs they took with the photographs and standard portion sizes in the photo atlas. The mothers were asked to name all ingredients used to prepare the food, and to identify its quantity, size, and weight in comparison with the examples in the photo atlas. The interview was delivered using the four stage, multiple-pass interviewing technique [23],[20]. Each child was followed for three consecutive days, and food intake was recorded using identical guidelines on each day of the study.

### Food database

Most of the nutrient data for individual nutrient calculations was derived from the USDA National Nutrient Database [24]. Data for individual food items was transferred and arranged into an Excel file. Some additional data was taken from Tanzania Food Compositions tables [25] and from Food Composition Tables developed for a specific area of Mozambique [26]. The decision to use primarily the USDA reference database was based upon the lack of available country food composition for Mozambique. Few leafy vegetables were unavailable in the food database; for these, closely related leafy vegetables from the same family (spinach, for example) were used, instead. Overall, the built database comprised 107 food items that were grouped and coded.

All foods, including local dishes, were arranged into 11 specific food groups. Each food item was arranged with a specific food code and number, depending upon food group. For example, staples such as maize, rice and bread represent carbohydrates; meat, eggs, fish, and legumes, represented a protein source. Potato and cassava represented the tubers consumed in the area. Leafy vegetables (lettuce, cowpea leaves, etc.) were placed in a different group than other vegetables (cucumber, tomatoes, etc.) due to difficulty in estimation, as their volume may not have represented their actual weight.

All food and amounts were collected in the Excel files, and the total amounts of energy and nutrient intakes were calculated. The selected nutrients included protein, fats, carbohydrates, calcium, iron, magnesium, zinc, phosphorus, folate, thiamin, niacin, riboflavin, and vitamins A, B, C, D, and E. These macro- and micro-nutrients were selected for their relevance to evaluate nutrient adequacies in children. Additionally, the intake of phytate was calculated based on the portion size of the various foods consumed and the phytate content of each food. Data of phytate content in the various food items was extracted from the FAO/INFOODS/IZiNCG Global Food Composition Database for Phytate [27]Additional data on phytates were taken from previous research on phytate and mineral content in plant-based food [28], [29]. The results of phytate and mineral intake were then used to calculate the molar ratios phytate-to-mineral. For the calculations, the molecular weights of 660 g/mol for phytate, 65.4 g/mol for zinc, 56 g/mol for iron, and 40 g/mol for calcium were used. Thereafter, the obtained molar ratios were compared with the recommended values for adequate absorption of zinc, iron and calcium.

### Statistical analysis

The normality of the data for all the parameters was evaluated by the Shapiro–Wilk test, and measurements of skewness and kurtosis. Most of the parameters did not follow normal distribution (p < 0.05). Therefore, the results are presented as median, minimum, and maximum values. Unpaired t-test was computed to evaluate significant differences between children in different age groups. Statistical analysis was completed using the Statistical Package for Social Sciences (SPSS), Version 22.0 (SPSS Inc., IBM Corporation 2013). The significance level was set at P < 0.05.

## Results

### Anthropometric measurements

A total of 56 children were followed, of whom 47 (84%) completed the study: 20 males and 27 females.19 were between 2–3 years old and 28 were between 4–5 years old (Table 1).

**Table 1:** Results of anthropometric measurements for the total group and for the subgroups 2–3 years old and 4–5 years old

	**Median**	**Minimum**	**Maximum**	**N (%)**	N (%) 2–3, Y*	N (%) 4–5, Y*
Age, Y	4	2	5			
Height, cm	97	71	116			
Weight, kg	15	7.6	23			
HA^1^ z-scores	−1.42	−6.83	1.35			
Stunting (HA < −2SD)				15(32)	10 (21.27)^a^	5 (10.63)^a^
Normal				32		
WA^2^ z-scores	−0.63	−3.67	1.67			
Wasted (WA < −2SD)				3(6)	3 (6)	0
Normal				44		
WH^3^ z-scores	0.34	−2.78	2.62			
Underweight/wasted (WH < −2SD)				1(2)	1(2)	0
Normal				40		
Overweight (WH > + 2SD)				6(13)	5 (10.63)	1(2.13)

^1^HA, Height-for-age, ^2^WA, Weight-for-age, ^3^WH, Weight-for-height

*Comparison between groups 2–3 years old vs. 4–5 years old. Different superscript letters indicate significant difference at *p* < 0.05

According to WHO classification [21], HAZ scores indicated that 32% of the children were stunted (short for projected height). WAZ scores indicated that 6% of the children were wasted (thin for their age), and WHZ scores indicated that 2% of the children suffered from wasting or underweight. Moreover, 13% of the children were overweight.

A strong negative correlation was observed between protein intake (*R* = −0.9), fat (*R* = −0.92) intake and wasting in children aged 2–3. A strong positive correlation was found between phytate intake (*R* = 0.99) and wasting. The more phytate the diet contents, higher levels of wasting were observed in the children. An inverse correlation was observed for 4–5 years old children between zinc intake and stunting (*R* = −0.55), suggesting that low zinc intake may be a factor that contributes to stunting in children.

### Description of dietary patterns

**Fig. 5:**
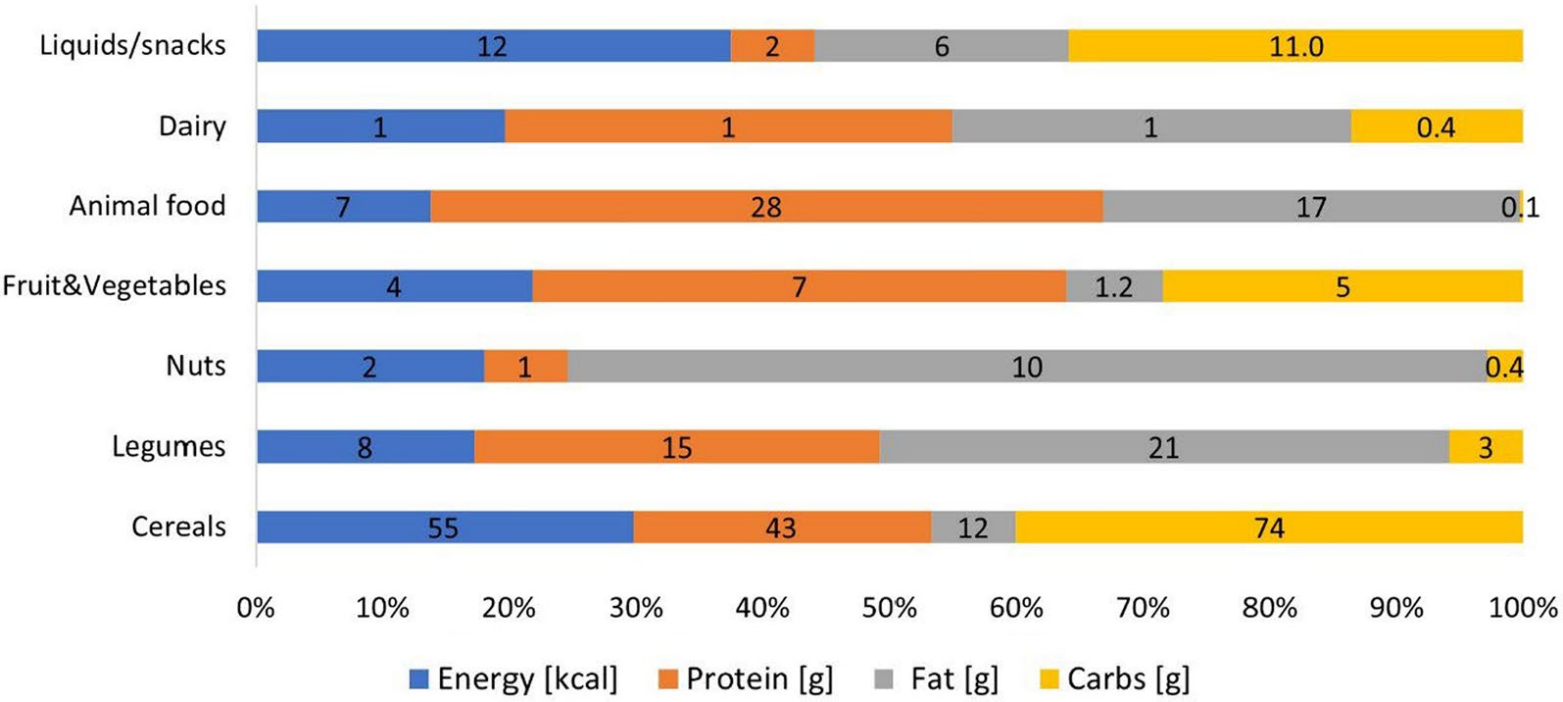
Contribution of different food groups to total energy and macronutrient intake

Dietary patterns (Fig. 5) were similar for the entire group of children (n = 47). Their diet was based mainly upon carbohydrates from cereals accounting up to 74% (rice 40%, maize 18%, bread 10%, pasta 6%); sugary drinks and snacks at 11%; tubers at 6% (potato 2%, sweet potato 4%); and legumes at 2% (beans). The highest proportion of protein consumed was plant-type, and came mostly from cereals, at 43% (rice 19%, maize 11%, bread 9%, pasta 5%); and legumes, at 15% (peanuts 10%, beans 5%). 25% of protein came from animal sources, mainly from fish (15%), chicken (8%), and some meat (2%). Eggs were rare in their diet and contributed to less than 3% of total protein consumed. Fat came mostly from vegetable oil (28%), peanuts (21%), coconut (10%), fish (8%), and, rarely, margarine (as low as 0.5%). Dark green leafy vegetables (cassava, cowpea, cabbage, or pumpkin leaves) were consumed frequently, at least once or twice during the three-day study period. Vegetables, generally tomato, onion, and green peppers were consumed in small portions, and usually used to make some kind of sauce. Consumption of seasonal fruit was very infrequent. Dairy was consumed extremely rarely, accounting for less than 1% of the children.

### Energy and nutrient intake

Dietary intake results (Table 2 and 3) for children were calculated in calories (kcal) for energy and the corresponding units for macro- and micronutrients. Because the recommendations for nutrient intake are different for children 2 to 3 years old than those 4 to 5 years old, results are presented for the two age groups separately. According to the dietary evaluation, the median energy intake was generally low: 674 kcal per day compared with the recommended energy requirements of 1310 kcal to 1690 kcal per day, depending on age and sex. The maximum energy intake was 1073 kcal per day, which did not reach the recommended values. The energy distribution was identical for both age groups. Compared with WHO’s recommendations, the energy distribution was within recommended values for protein, contributing to 16%E of total energy and carbohydrates contributed to 75%E. In contrast, the intake of fat was very low, and contributed to only 12%E of total macronutrients, which is below the recommended value of 25%—40% total energy [30]. The dietary intake of carbohydrates, protein, magnesium, thiamin, riboflavin, niacin, vitamin C, and vitamin B6 were more than 82% of the RDA (Recommended Dietary Allowance) for both age groups.

**Table 2:** Daily average nutrients intake of children 2–3 years old

Nutrient	Median	Minimum	Maximum	%RDA^2^ Met by the diet n = 19
Energy Kcal/day	593.6	292.3	1073.2	
Protein g/d	16.54	9.7	33.1	131
Carbohydrate g/d	90.37	43.6	178.8	82
Fat g/d	16.05	7.7	30.0	
Fiber g/d	6.61	2.2	16.21	
Calcium mg/d	81.38	30.8	365.3	12
Iron mg/d	6.4	2.9	10.5	101
Magnesium mg/d	100.35	44.88	215.81	141
Zinc mg/day	2.36	1.1	8	80
Phosphorus mg/day	277.4	133	477.6	64
Vitamin A µRAE/d	116.76	28.47	763.49	49
Vitamin C mg/d	22.17	4.66	119.17	188
Vitamin E α-Toc mg/d	2.22	0.7	12.6	45
Thiamin mg/d	3.16	0.76	14.14	656
Riboflavin mg/d	1.33	0.42	4.66	278
Niacin ug/d	4.99	1.75	14.84	96
Vitamin B-6 mg/d	1.92	0.35	6.90	384
Folate ug/d	84.72	32.39	174.57	59
Vitamin B-12 ug/d	0.19	0	1.22	41
Vitamin D ug/d	0.52	0	3.43	3.5
Phytates mg/d	0.67	0.25	2.02	
Molar ratio phy:Zn^1^	29.97	8.05	59.58	
Molar ratio phy:Fe^1^	8.87	5.54	25.777	
Molar ratio phy:Ca^1^	0.49	0.11	2.19	

^1^Suggested molar ratios of phy:Ca < 0.24, phy:Fe < 1 and phy:Zn < 15; above which the bioavailability of these minerals may be inhibited by phytate content in the diet. The molar ratios were determined by dividing the weight of phytate and minerals by their atomic weight. Phytate:mineral molar ratio obtained by: Phytate(mol)/Mineral(mol)

^2^%RDA, percentage of Recommended Dietary Allowance, calculated as: %RDA = (Estimated nutrient intake from the diet/Recommended nutrient intake for children aged 1–3 yo)*100

**Table 3:** Daily average nutrients intake of children 4–5 years old

Nutrient	Median	Minimum	Maximum	%RDA^2^ Met by the diet n = 28
Energy Kcal/day	695	439.6	1016.4	
Protein g/d	18.3	11.1	34.3	89
Carbohydrate g/d	115.36	70.2	165	82
Fat g/d	18.76	8.6	30.3	
Fiber g/d	8.86	4.4	22.9	
Calcium mg/d	89.3	44.1	157.8	9
Iron mg/d	7.8	4.5	10.3	71
Magnesium mg/d	122.8	67.91	199	88
Zinc mg/day	2.6	1.7	4.7	48
Phosphorus mg/day	306.7	200.5	508.4	59
Vitamin A µRAE/d	176.3	12.43	2458.76	37
Vitamin C mg/d	27.5	7.51	88.20	113
Vitamin E α-Toc mg/d	3.1	0.8	5.9	39
Thiamin mg/d	3.3	1.05	18.74	547
Riboflavin mg/d	1.4	0.59	5.38	232
Niacin ug/d	6.5	2.59	11.63	72
Vitamin B-6 mg/d	2.1	0.49	5.79	320
Folate ug/d	96.7	45.21	163.17	44
Vitamin B-12 ug/d	0.5	0	18.30	31
Vitamin D ug/d	1.4	0	15.73	3.5
Phytates mg/d	0.8	0.310	1.836	
Molar ratio phy:Zn^1^	25.7	8.42	57.85	
Molar ratio phy:Fe^1^	8.2	3.16	22.63	
Molar ratio phy:Ca^1^	0.54	0.16	1.34	

^1^Suggested molar ratios of phy:Ca < 0.24, phy:Fe < 1 and phy:Zn < 15; above which the bioavailability of these minerals may be inhibited by phytate content in the diet. The molar ratios were determined by dividing the weight of phytate and minerals by their atomic weight. Phytate:mineral molar ratio obtained by: Phytate(mol)/Mineral(mol)

^2^%RDA, percentage of Recommended Dietary Allowance, calculated as: %RDA = (Estimated nutrient intake from the diet/Recommended nutrient intake for children aged 4–8 yo)*100

Table 2 and 3 also present the micronutrients intake and adequacy for the two different age groups respectively. Of the recommended values, intake of calcium and vitamin D were very low in both groups; children 2–3 years old met only 12% for calcium and 3.5% for vitamin D, and children 4–5 years old met only 9% for calcium and 3.5% for vitamin D. Vitamin A intake was also below the recommended value, meeting the requirements by only 49% for younger group and 37% for older group of children.

### Phytate intake and estimated mineral bioavailability

Iron intake met 101% of the iron-RDA for children aged 1–3 years, but only 71% for those aged 4–8 years. Zinc intake met 80% of the zinc-RDA for children aged 1–3 years and 48% for children over 3 years. Intake of folate, vitamin E, and vitamin B12 ranged from 31 to 59% of the recommended amounts, depending on the age group. Additionally, intake of phytate and phytate-to-mineral molar ratios were evaluated, providing the ratios of phy:Zn between 8.5 and 59; phy:Fe between 3.2 and 25.8; and phy:Ca between 0.11 and 2.19. Micronutrient adequacy was compared between both groups, revealing a statistically significant difference (*p* < 0.05) in the intake of iron and vitamin A, with lower levels of adequacy observed in children aged 4–5 years.

## Discussion

### Anthropometric measurements

In the present study the nutritional status of children aged 2 to 5 years in the rural area Bobole in Mozambique was assessed. The z-scores of HAZ, WAZ and WHZ have shown that 32% of children were stunted, 6% were wasted, 2% were underweight, and 13% of children were overweight. The prevalence of stunting is a significant concern in Mozambique, as it highlights the persistent, long-term structural issues contributing to undernutrition. However, stunting prevalence showed improvement decreasing from 42.6% in 2012 to 36.4% in 2022. This reduction aligns with the findings reported by Shipanga et al. [2]. Notably, in this study the percentages of stunted and wasting in children were lower than reported in the previous study done in Zambézia Province in Mozambique, where prevalence ranges of stunting and underweight were 49 – 70% and 14–24% respectively for children aged 6–35 months [31]. This suggests regional differences in nutritional outcomes and potential improvements over time. The reason for stunting in children can be associated with the low energy intake, ranging from as low as 292 to a maximum of 1073 kcal per day. While the recommended average energy intake is about 1350 kcal for children 2—4 years of age, and about 1560 kcal for children 4—5 years of age, depending on the age and activity level [30].

Additionally, the high percentage of stunted children may be related to zinc deficiencies. In this pilot study, an inverse correlation was observed between zinc intake and stunting (*R* = −0.55). It has been previously reported that zinc is a limiting nutrient in children’s diet, with deficiencies leading to progressive stunting, impaired immune system and abnormalities in body composition. More specifically, zinc, which is an essential trace element, plays a critical role in the synthesis of body proteins, cellular growth, and differentiation. Growth failure in children from rural areas in developing countries has been associated with multiple factors, particularly monotonous and imbalanced diets due to a lack of education and/or socioeconomic factors. Furthermore, infections including viral, bacterial, and intestinal infections may create a vicious cycle between poor nutrition and illness, further hindering children’s ability to achieve normal growth and development, as shown in previous research of Lazarte et.al. [32].

Further, a strong positive association was found between phytate intake and wasting in the observed children. However, these results should be interpreted with caution, as this was a pilot study of a limited sample size. Nevertheless, the findings suggest that further studies with larger sample sizes and expanded study areas could provide more robust evidence on the effects of dietary phytate on growth and development. Phytate, commonly found in plant-based foods such as cereals, legumes, and seeds, has been found to significantly inhibit the absorption of protein and essential minerals like zinc and iron. This condition is especially concerning in populations with limited dietary diversity, where high-phytate foods form a large part of the diet, and alternative sources of highly digestible protein or nutrient-rich foods are unavailable. The mechanism by which phytate inhibits nutrient absorption in the gastric system is primarily attributed to its strong ability to bind positively charged molecules, such as small proteins and minerals, forming insoluble complexes. These complexes are not readily broken down in the human digestive tract, making the bound nutrients unavailable for absorption, and excreted out of the organism without being utilized.

About 13% of children were overweight, which aligns with South African Demographic and Health Survey (SADHS) conducted in 2016, it was indicated that 13% of children under five were overweight [33]. Overweight does not indicate that these children have a better nutritional status; in fact, they may lack essential vitamins and minerals even though they consume a sufficient number of calories. This phenomenon is called “hidden hunger”. Since the signs of undernutrition are not that visible in many cases, people may not be aware of the situation [34]. It was reported that the increased occurrence of overweight children in poor regions is a result of “nutrition transition” which includes qualitative and quantitative changes in the diet. These changes usually include the shift of the diet towards reduced fruit and vegetable intakes, to a greater energy dense foods, acquired from saturated fats, refined carbohydrates and added sugars in foods [35].

### Dietary patterns and nutrient adequacy

It is indicated that dietary diversity can be a strong indicator of dietary quality and micronutrient adequacy in children [36]. In the present study, the nutrient contribution from different food groups **(**Fig. 5**)** was dominated by starchy staples, with little day-to-day variation. The most common staples consumed were rice, maize and bread, contributing to 55% of the total energy intake. Cereals provided almost half (43%) of the protein, and more than half of iron, magnesium, phosphorus and vitamin A as well as over 40% of thiamin and niacin and quarter of folate in these diets. The consumption of leafy greens (mainly from cassava, pumpkin, sweet potato, cowpea, local plant “cacana”, and cabbage leaves), legumes and nuts in this population provided essential nutrients. The traditional leafy green stews, prepared with peanut or coconut, provided over 20% of calcium, vitamin A, and vitamin E. Legumes and nuts were the second largest source of fat, zinc, iron, folate, magnesium, riboflavin and vitamin E after starchy staples play an important role in supplementing the diet with essential nutrients. Although animal-based foods were consumed rarely in relatively very small quantities, they provided over a quarter of total protein and vitamin A and nearly all vitamin B12 and vitamin D in the diet. These foods were also important sources of fat and niacin in the diet. Dairy products were almost not consumed in the area, mainly because of purchasing costs, which may be prohibitive for some families. Consumption of snacks and sugary drinks was common and contributed to 11% of total carbohydrates. Studies conducted, with the same dietary assessment method in rural areas of Bolivia have shown that the primary source of carbohydrates was cereals, tubers and legumes, while protein intake from meat or egg was limited [37]. Furthermore, similar dietary patterns were also observed in studies conducted in few other rural areas of Mozambique, where an even more energy came from starchy staples (82%) and only 2% of protein was derived from an animal-based food [31, 38].

The surprising finding of this study was the protein intake. The median intake was estimated to be 17 g of protein, contributing 13% of total energy, which falls within the recommended range of 5–20% of total calories, being contributed by protein, for children 1—3 years and 10–30% for children 4 to 8 years of age [30]. Thus, these results indicated an adequate protein consumption in the investigated group. These results are positive as the nutritional effects of low protein intake can lead to protein deficiency and malnutrition, while also high protein intake during early childhood have been reported to elevate the risk of developing adiposity later in life due to increase secretion of insulin [39]. In contrast, median fat intake was very low, at 19 g, contributing only 12% of total energy intake, which is below the range of recommended values, 30–40% of total calories for children 1—3 years of age and 25–35% of total calories for children 4 to 8 years of age should come from fat [30]. The low fat intake reported here is comparable to previous studies in rural Mozambique [38] and Bolivia [32] where diets tend to be low in fats particularly those from animal sources (17%), generally fish and poultry. The remaining fat came from legumes (21%), starchy staples (14%) and nuts (10%). This distribution suggests that while plant-based fats are present in the diet, they may not provide enough to meet recommended intake levels. Although legumes, starchy staples, and nuts provide beneficial fats, they may not offer the full diversity of fatty acids found in animal sources, which are essential for growth and brain development in children [40]. This highlights the need for further research on the role of essential fatty acids in the diets of children in rural areas of developing countries.

The largest proportion of dietary intake was taken over by carbohydrate, median intake of 106 g per day, contributing to 74% of total energy intake of the children which is above the recommended energy distribution range of 45–65% [30]. Similar results were found in the previous studies in Mozambique [31, 38] and Bolivia [32]. Such dietary patterns are typical in rural areas or in households where mothers have lower levels of education, limiting access to a more diverse diet [41]. According to our estimates, only about 5% of carbohydrates came from nutritionally rich sources such as fruit and vegetables, although fruit consumption is difficult to estimate because children often play outside the house and pick fruits directly from trees to eat. Another noteworthy concern is the contribution of snacks and added sugar to the children’s diet. Approximately 11% of carbohydrates and 12% of total energy came from snacks like chips, popcorn and added sugars. The consumption of added sugar is particularly problematic as it contributes to energy intake without providing essential nutrients, potentially reducing the nutrient density of the diet.

The median iron intake in the study was 7.1 mg/day meeting 101% of the recommended daily allowances for children aged 2—3 years and 71% for children aged 4 to 5 years. These results align with findings from a study conducted in other rural areas [32]. However, despite the adequate overall intake, the quality of iron intake raises concern. Only about 6% of iron came from animal food sources. Heme iron found in animal products is more bioavailable and has an absorption rate of approximately 25% whereas non-heme iron present in plant-based foods, dairy and eggs is absorbed at a significantly lower rate (< 10%). This distinction is crucial because even though total iron intake seems to be adequate, the low intake of bioavailable heme iron could leave children at risk of iron deficiency. This concern aligns with findings from UNICEF, which reports that iron deficiency or anemia affects 75% of children in Mozambique [42]. Iron absorption in the diets of these children may be inhibited by phytate in their diets. Phytate is a compound commonly found in cereals and legumes, which are the main dietary staples in many LMICs. The inhibitory effect of phytate on iron absorption depends on phy:Fe molar ratio. In this study the molar ratios phy:Fe in the diets ranged between 3.2 and 25.8, exceeding the recommended threshold for optimal iron absorption (phy:Fe < 1).

The intake of vitamin A was notably low, median of 146 RAE/d with a minimum intake as low as 12 RAE/day. This is far below the recommended dietary allowance of 300 RAE/day for children aged 1–3 years and 400 RAE/d for children aged 4—8 years. The diets of the children in this study provided only 49% of the recommendations for vitamin A in the younger group (2—3 y) and 37% in the group of children aged 4–5 years. Furthermore, only 21% of the children’s diet met the recommended intake for vitamin A, indicating that 79% of them may be at risk of vitamin A deficiency. These findings align with data from UNICEF, which indicate that 69% of children under five in Mozambique suffer from vitamin A deficiencies. Vitamin A deficiency is a major manifestation of malnutrition in Mozambique, often resulting in severe health consequences, including irreversible corneal damage and an increased risk of partial or total blindness [43]. Despite 25 years of biannual vitamin A supplementation programs, improvements in vitamin A deficiency have been modest, with coverage increasing from 44% in 2003 to just 55% in 2017 [44].

In this study it was estimated that the median zinc intake was 2.4 mg/day. The diet met 80% of the recommendations for zinc in children aged 2 to 3 years and 48% of the recommendations for children aged 5–6 years. Dietary zinc intake reached about 80% of recommended values in only one-quarter of the children, suggesting that about 75% of the children are at risk of zinc deficiency. The results are consistent with findings from various studies conducted on children in rural regions, including Bolivia [32], India and Bangladesh, where similar risks of dietary deficiencies were observed [45] [46]). Most dietary zinc was obtained from cereals (52%), legumes (13%) and animal food sources (12%). Zinc is a crucial nutrient for growth and development, particularly in populations with high prevalence of malnutrition, where it is often a limiting growth nutrient. A meta-analysis of 43 studies examining the impact of dietary zinc interventions on growth revealed a 61% positive effect on growth outcomes [47]. While cereals and legumes appear to supply sufficient amounts of zinc, they also contain phytates, which is a major inhibitor of zinc absorption and availability [30]. Consequently, the actual amounts of zinc absorbed may be considerably lower than the estimated intake.

The most extreme micronutrient inadequacy in the diet was calcium, with a median intake of 89 mg per day. Calcium intake met only the 12% of the RDA for children aged 2—3 years and 9% of RDA for children aged 5—6 years. The intakes were very low considering the importance of calcium in bone development for rapidly growing children. A similar calcium intake inadequacy (80.1%) was obtained in a 24 h-recall study evaluating the nutritional status of mother–child pairs aged from 6–24 months in Malema and Gurue districts in northern Mozambique [48]. Calcium may not directly influence longitudinal growth; it is essential for maintaining adequate bone density. Numerous studies have linked calcium deficiency to the development of rickets in toddlers and children, a condition closely associated with vitamin D deficiency. Low dietary calcium intake results in increased catabolism of vitamin D and therefore development of rickets [49, 50]. The obtained calcium intake results are similar to previous studies [51], which have reported that diets in developing countries are generally low in calcium. Furthermore, the actual calcium absorption may be even lower than dietary intake, since most of calcium came from plant-based sources. Compared to the calcium absorption from milk, only about half of the calcium from legumes is absorbed, and only one-tenth is absorbed from leafy greens like spinach [30].

Dietary intakes of folate showed a median of 88 µg/day, this intake met only 59% of the RDA for children aged 2—3 years, and 44% of the RDA for children 5 to 6 years of age. A similar folate dietary inadequacy (29%) was reported in children aged 1–5 years who were HIV infected in Uganda [52]. In our study, most of the dietary folate was acquired from legumes (35%), fruit and vegetables (54%) and cereals (22%). The actual dietary folate may be less bioavailable for absorption than the estimated intake, as food folate is typically only 30% to 80% as efficient as folic acid, the synthetic form added to food or found in supplements. Folate plays an important role in the metabolism of nucleic and amino acids and its deficiency may lead to decrease in red blood cells and megaloblastic anemia [30].

### Phytate intake and estimated mineral bioavailability

The molar ratios of phytate to minerals are commonly used to estimate the extent to which phytate inhibits mineral absorption. According to our study, phytate was predominantly contributed to the children’s diets, from staples such as cereals, legumes, and in lower amounts from roots and tubers. Over 45% of phytate was found in rice and maize, and about 40% of phytate was found in legumes. Since children’s diets were dominated by staple foods, it is likely that these diets provided a low bioavailability of zinc, iron and calcium, due to the phytate content and its inhibitory effect on these minerals. According to our study phy:Zn molar ratios in diets ranged between 8.5 to 59. The ratios are similar to the ones reported in the study done in children living in other rural areas [32]. The IZiNCG consultative group [17] has indicated that phy:Zn molar ratios above 15, indicate that the absorption of zinc may be inhibited by the presence of phytate. Further, according to the WHO committee, in the diets mainly based on whole cereal grains or flours rich in phytate and vegetables, the absorption of zinc may only reach 15% to 30% when for phy:Zn are above 15 and for phy:Zn between 5 and 15 respectively. In this pilot study 89% of the children’s diets presented phy:Zn molar ratios above 15, suggesting that the bioavailability of zinc in those diets may be declined to as low as 15% of the zinc content in the diet. Only 15% of the zinc content in those diets may be absorbed and utilized by the body functions. Moreover only 11% of the children’s diets presented phy:Zn ratios between 5 and 15 suggesting that zinc bioavailability of those diets was as low as 30%, and none of the children’s diets presented phy:Zn ratios below 5, which is the level where phytate will unlikely inhibit the absorption of zinc in the diet. When the diet is mixed however, the influence of phytate on absorption of zinc depends on the overall composition of the diet, where the presence of animal protein can improve zinc absorption and the presence of calcium may inhibit zinc absorption, due to the formation of insoluble calcium-zinc-phytate complexes from the phytate rich diets [53]. There has been a great interest and numerous studies have been reported on phytate and its effects on zinc bioavailability, suggesting that the presence of phytate in the diet may lead to zinc deficiencies [32], [53].

Likewise, the molar ratios of phy:Fe in the diet of the children were also higher than anticipated, ranging between 3.2 and 25.8. It is suggested that when phy:Fe ratio is above 1, the bioavailability of iron may be strongly inhibited by the phytate. The phy:Fe ratios in the present study were quite high, however, it has been suggested that the inhibitory effect of phytate on iron absorption is related to the degree of the metal saturation of the phytate-iron complexes present in foods and other contents of the diet [53]. The results of the present study were similar to previous studies where phy:Fe molar ratio exceeded 1 in all participants [32].

Moreover, phy:Ca molar ratios (0.11 to 2.19) were also higher than the recommended value of 0.24, suggesting that absorption of already extremely low intakes of calcium may be further compromised by the phytate content in the diet. Previous studies have reported, that consumption of foods rich in phytate inhibit the absorption of calcium from the intestines due to the chelation of phosphate groups in phytate with divalent cations of calcium, making it harder for calcium to be absorbed through the intestinal lining [54]. Consequently, children who consume diets rich in phytate and have low calcium intakes may be more prone to calcium deficiency than estimated, which can negatively affect the bone mineral density and overall bone health of the children.

The amount of phytate and its inhibitory effect on the absorption of essential minerals can be reduced through various traditional processing methods, such as soaking, fermentation, germination, as well as thermal and mechanical processes, all of which can make the minerals more bioavailable in the diet [54]. Ultimately, diets of children who consume very low or no animal food sources should aim to achieve lower phytate levels. This can be accomplished by avoiding unrefined cereals and legumes high in phytate and by using food processing methods that reduce phytate content.

### Children’s dietary inadequacy in the context of Mozambican food environments

The dietary patterns and micronutrient inadequacies observed are deeply intertwined with the broader context of food insecurity and food environments in Mozambique. Food insecurity remains a significant challenge, particularly in rural areas where poverty rates are high and access to diverse, nutrient-rich foods is limited [55]. The reliance on starchy staples and the lack of dietary diversity seen in this study reflect the food insecurity prevalent in Mozambique. Households often depend on readily available and affordable staples like rice and maize because economic constraints limit access to a wider variety of foods [56]. Economic hardship is a key factor influencing food environments, which can be referred as an interface that connects consumers to the food system, where the consumers can make choices about sustainable, accessible, affordable, convenient and desirable diet [57]. In pre harvest period where many households fall into low dietary diversity in Mozambique, the failure to achieve adequate food environments is more pronounced [58]. This is due to the fact that households have low purchasing power, and they feed the children with what is available in the season and often in smaller amounts than required. In general, the available staples are rice and maize, and the dependence on seasonal staples can be conducive to nutrient deficiencies among children. Other factors that contribute to food insecurity are low level of education, informal work [55] age and gender of the household head in Mozambique [58].

In communities with poor resources malnutrition arises not only from insufficient food quantities but also from the inadequate nutritional quality of the available food supply [59]. This issue is particularly pronounced in plant-based diets, which often contain minimal amounts of micronutrient-rich animal-source foods. Additionally, the presence of antinutrients such as phytate, polyphenols, and oxalates contributes to the low bioavailability of essential minerals, further compromising the quality of predominantly plant-based diets [23]. This problem is particularly acute in diets lacking animal-source foods, where nutrients from plant-based staples become less bioavailable, exacerbating micronutrient deficiencies [60]Given that low-income populations heavily rely on cereals as a primary food source, the adverse effects of low mineral bioavailability on mineral status and overall health can be significant.

To address these challenges, a range of interventions tailored to the needs of rural communities must be explored to enhance both food access, quality and nutritional outcomes. Such interventions could involve promoting dietary diversity, utilizing phytate-reduction methods or fortifying food staples to improve overall dietary bioavailability and address both the quantity and quality of available nutrition. Several traditional household food-processing and preparation methods can be used to enhance the bioavailability of micronutrients in plant-based diets, which include thermal processing, mechanical processing, soaking, fermentation, and germination/malting [17, 31]. These strategies aim to increase the physicochemical accessibility of micronutrients, decrease the content of antinutrients such as phytate, or increase the content of compounds that improve bioavailability. A combination of strategies is probably required to ensure a positive and significant effect on micronutrient adequacy.

### Limitations of the study

We present the results of a pilot study conducted in the rural area of Bobole, Mozambique. While the study provides valuable insights, it faces limitations primarily due to the relatively small number of participants, which may reduce the generalizability of the findings to the broader population. A smaller sample size constrains statistical power, increasing the likelihood of missing significant dietary patterns or associations. Moreover, the variability in dietary habits across diverse rural communities may not be fully represented. Despite these limitations, the use a validated dietary assessment method such as FP24hR enhances the reliability and accuracy of the collected data, ensuring that the findings, while limited in scope, are robust within the study’s context. These results offer a foundation for future research and contribute important insights into dietary behaviours in populations in rural areas of LMIC.

To build on this work, we recommend replicating the study with a larger number of participants and extending it to include a broader range of rural areas. Future studies could also benefit from improvements such as expanding the nutrient calculation database and increasing the diversity of ingredients and foods illustrated in the photo atlas used for portion size estimation. Additionally, incorporating biochemical markers, such as serum concentrations of iron and zinc, could provide more accurate and comprehensive data on nutritional status. Furthermore, this pilot study revealed differences between two groups of children of different ages, highlighting the need for further research to explore how eating habits and behaviors evolve during the transition from toddlerhood to the preschool years.

## Conclusion

Anthropometric measurements and dietary data were collected from 47 children, ages 2 through 5, living in rural area Bobole in Mozambique. The most important findings showed that 32% of children were stunted, 6% of children were wasted, 2% were underweight and 13% were overweight. High prevalence of stunting indicates failure in growth, suggesting that there is a sustained problem of child undernutrition in the area. The median zinc intake was less than 50% of the RDA and the dietary phytate was found to be high in the diets, which may be key factors contributing to growth failure. Moreover, the estimated bioavailability of zinc, iron and calcium was found to be likely diminished by the phytate content in the children’s diets. Future nutritional intervention strategies will be useful to prevent malnutrition and stunting among children is rural areas of Mozambique and other LMICs.

The results of this study highlight the crucial connections between dietary patterns, micronutrient inadequacy, and mineral bioavailability in children, linked to the broader challenges of food insecurity and food environments in Mozambique. Addressing these issues will require integrated strategies focused on improving agricultural practices, increasing access to fortified foods, enhancing nutrition education, and implementing supportive food policies to ensure that vulnerable populations, especially children, have access to a diverse and nutritionally adequate diet.

## Data Availability

All data that support the findings of this study are available upon request.

## Abbreviations

HA: Z-score height-for-age
WA: Z-score weight-for-age
WH: Z-score weight-for-height
phy Ca: Molar ratio phytate to calcium
phy Fe: Molar ratio phytate to iron
phy Zn: Molar ratio phytate to zinc
